# Impact of the Affordable Care Act Medicaid expansion on oral surgery delivery at community health centers: an observational study

**DOI:** 10.1186/s12903-021-01895-4

**Published:** 2021-10-20

**Authors:** Evan V. Goldstein, Wendy Yi Xu, Eric E. Seiber

**Affiliations:** 1 Department of Population Health Sciences, School of Medicine, University of Utah, Salt Lake City, UT 84108 USA; 2 Division of Health Services Management and Policy, College of Public Health, Ohio State University, Columbus, OH 43210 USA

**Keywords:** Primary care, Access to care, Medicaid, Oral surgery

## Abstract

**Background:**

Unmet oral health needs routinely affect low-income communities. Lower-income adults suffer a disproportionate share of dental disease and often cannot access necessary oral surgery services. The Affordable Care Act (ACA) Medicaid expansion created new financial opportunities for community health centers (CHCs) to provide mission-relevant services in low-income areas. However, little is understood in the literature about how the ACA Medicaid expansion impacted oral surgery delivery at CHCs. Using a large sample of CHCs, we examined whether the ACA Medicaid expansion increased the likelihood of oral surgery delivery at expansion-state CHCs compared to non-expansion-state CHCs.

**Methods:**

Exploiting a natural experiment, we estimated Poisson regression models examining the effects of the Medicaid expansion on the likelihood of oral surgery delivery at expansion-state CHCs relative to non-expansion-state CHCs. We merged data from multiple sources spanning 2012–2017. The analytic sample included 2054 CHC-year observations.

**Results:**

Compared to the year prior to expansion, expansion-state CHCs were 13.5% less likely than non-expansion-state CHCs to provide additional oral surgery services in 2016 (IRR = 0.865; *P* = 0.06) and 14.7% less likely in 2017 (IRR = 0.853; *P* = 0.02). All else equal, and relative to non-expansion-state CHCs, expansion-state CHCs included in the analytic sample were 8.7% less likely to provide oral surgery services in all post-expansion years pooled together (IRR = 0.913; *P* = 0.01).

**Conclusions:**

Medicaid expansions can provide CHCs with opportunities to expand their patient revenue and services. However, whether because of known dental treatment capacity limitations, new competition, or coordination with other providers, expansion-state CHCs in our study sample were less likely to provide oral surgery services on the margin relative to non-expansion-state CHCs following Medicaid expansion.

## Background

Over $45 billion in productivity may be lost each year in the US because of untreated oral disease [1]. Unmet oral health needs especially affect low-income communities in which federally-funded community health centers (CHCs) operate [2], serving as barriers to employment [3] and educational progress for many patients [4]. Low-income adults suffer a disproportionate share of dental disease [5], and about 43.9% of non-elderly low-income adults have untreated tooth decay [6], often requiring surgical treatment like extraction [7]. In response, many CHCs seek to eliminate barriers to dental services historically caused by a separation from the broader medical care system [8]. Over 80% of CHCs provide some dental services [9]. However, treatment capacity is often limited [10], and many CHC patients struggle to access necessary dental care [11]. Although CHCs provide uncompensated dental services to uninsured adults, dentistry is often not free for CHC patients [12]. CHCs are often limited by how much uncompensated dentistry they can provide [12].

Predating the Affordable Care Act (ACA), about 33% of non-elderly adults had no dental insurance [13]. Consequently, the ACA Medicaid expansion provided new opportunities to CHC patients to gain dental coverage and to CHCs to expand patient revenue and service capacity. Revenue from Medicaid-covered patient visits can help CHCs offset the cost of providing oral surgery and other dental services as Medicaid payments reasonably approximate the cost of care for CHC encounters, typically more than other payers [14]. Prior to the full implementation of the Medicaid expansion, other studies predicted that the Medicaid expansion could cause an inflow of new Medicaid-covered adults with dental benefits and increase the demand for dental care in states that cover adult dental services through Medicaid [15]. On the other hand, Reynolds et al. [10] expressed concerns that CHCs would be limited in their ability to implement the changes needed to meet increased demand for dental services as a result of the expansion. In the non-dental-care context, previous studies have shown that the ACA Medicaid expansion led to increases in Medicaid-covered medical care appointments at CHCs [16, 17] and increased patient Medicaid revenue by 97% from 2010 to 2017 [18].

Notably, states also have discretion in defining adult Medicaid dental benefits, and not all states provide reimbursement for comprehensive dental benefits through Medicaid. One ACA Medicaid expansion state (AZ) covers no adult dental services [19], and other expansion states provide emergency-only or limited benefits [20]. Nevertheless, in states that do provide adult dental benefits, expanded Medicaid eligibility and patient revenue opportunities may have helped CHCs expand dental treatment capacity in their clinics or through coordination with community practitioners. Little is understood in the scholarly literature, however, about how the ACA Medicaid expansion affected oral surgery delivery at CHCs, despite the importance of these services for populations experiencing lower incomes.

### Research objective

Our objective was to examine whether the Medicaid expansion increased the likelihood of oral surgery delivery at expansion-state CHCs relative to non-expansion-state CHCs. Examining a large sample of CHCs, we compared the quantity of oral surgery services delivered at expansion-state CHCs and non-expansion-state CHCs from 2014 to 2017. Because previous studies suggest new Medicaid policies may take time to affect oral health care utilization for different patient populations [21–23], we hypothesized that the Medicaid expansion would increase the likelihood of oral surgery delivery at expansion-state CHCs relative to non-expansion-state CHCs, but that the effects of the Medicaid expansion would be greater at the end of the study period.

## Methods

### Data

Our primary data source was the uniform data system (UDS) for the period 2012–2017 (calendar years from January 1 to December 31), accessed through Freedom of Information Act requests (#19F122 and #19F270) [24]. The Health Resources and Services Administration (HRSA) collects the UDS data annually on CHCs’ patient characteristics, service utilization, and organizational features. For purposes described below, we also used data from additional sources, including the Kaiser Family Foundation [25], Bureau of Labor Statistics (BLS) [26], Behavioral Risk Factor Surveillance System (BRFSS) [27], and the Current Population Survey (CPS) through IPUMS CPS [28].

### Sample

Exclusions were made attempting to ensure included CHCs experienced similar policy exposure and implementation efforts for similar amounts of time [16, 29]. Consistent with other studies [15], we included CHCs from states that covered the expenses for oral surgery services through their state Medicaid programs for non-elderly adults during the study period [20, 30, 31]. CHCs in US territories were excluded, as were CHCs from six states (CA, CT, MN, NJ, WA, and DC) that expanded Medicaid in 2014, but also for some residents prior to 2014 (prior to the start of the study data) [16, 32]. Lastly, CHCs from states that expanded Medicaid during the study period but after 2014 were excluded. This exclusion was made to avoid empirical concerns about the influence of variation in policy exposure timing on the estimated policy effects [33], and to focus on examining the initial and intermediate effects of the Medicaid expansion on previously-studied CHCs operating in states that expanded at first opportunity in 2014.

The study included 340 unique CHCs per year. The CHC-year was the unit of analysis, and our analytic sample included 2054 observations from 17 states (AR, CO, IA, IL, MA, NC, NE, NM, NY, OH, OR, RI, SC, SD, VT, WI, WY), including a Medicaid expansion policy group of 1572 expansion-state CHC-year observations (AR, CO, IA, IL, MA, NM, NY, OH, OR, RI, VT) and a comparison group of 480 non-expansion-state CHC-year observations (NC, NE, SC, SD, WI, WY).

### Analysis

We exploited natural variation between CHCs in expansion (policy group) and non-expansion (comparison group) states before and after the ACA Medicaid expansion in 2014. In the first stage of our analysis, we estimated an event study model to examine changes in the relative oral surgery delivery outcomes between the policy and comparison groups while adjusting for observable differences between the two groups and fixed differences across states and over time. For this stage of the analysis, the coefficients of interest were those for interaction terms between a Medicaid expansion variable and variables indicating the time relative to the Medicaid expansion adoption year. In a second stage of the analysis, we calculated a difference-in-differences estimate as a summary of the Medicaid expansion policy effect across the post-expansion years. The coefficient of interest in the second model was a single variable denoting an expansion state during the post-expansion period. All model variables are discussed in detail below.

This analytic approach assumed that, absent the Medicaid expansion, the average changes in the outcomes would have been the same for both the expansion-state and non-expansion-state groups. A corollary of this untestable common trends assumption was examined statistically in the event study model results [34, 35].

Robust standard errors were clustered at the policy intervention (state) level to correct for heteroskedasticity and serial correlation [36]. Because the outcome variable was measured as a count of oral surgery services, we estimated multivariate Poisson regression models. For ease of interpretation, the coefficients are presented as incidence rate ratios (IRRs). All analyses were conducted using Stata version 17.1.

### Outcome variable

Our outcome variable measured the number of oral surgery service visits at a CHC in a year, including extractions and other surgical procedures, identified using Code on Dental Procedures and Nomenclature (CDT) codes D7111, D7140, D7210, D7220, D7230, D7240, D7241, D7250, D7260, D7261, D7270, D7272, D7280.

### Explanatory variables

To examine the effect of the Medicaid expansion on our outcomes, we used Kaiser Family Foundation data to construct a binary variable indicating whether the Medicaid expansion was adopted in a state [25]. For the first stage of the analysis, we interacted the Medicaid expansion status variable with binary variables indicating the time relative to the Medicaid expansion adoption year (i.e., 2014) to examine whether the estimated effect of adopting the expansion on oral surgery services delivery increased or decreased in the years following expansion. For the second stage of the analysis, we replaced the event study indicators with a single variable denoting a Medicaid expansion state during the post-expansion period, which switched on starting in 2014.

### Covariates

Our statistical models included a vector of time-variant covariates to absorb residual variance in the outcomes or adjust for potential confounding factors, especially organizational and patient population differences between the policy and comparison CHC groups. UDS data were used to adjust for CHC-level patient factors, including the gender, race/ethnicity, age, and income compositions (< 100% of the Federal Poverty Level) of CHC patients. Annual percentages of patients diagnosed with depression/mood disorder and diabetes mellitus were included to account for patient health status and potential need for treatment differences between CHCs, as depression and diabetes mellitus are associated with poor oral health [37–40]. A measure of total patient population was included to adjust for differences in organizational size and capacity, as well as the assumption that practices with more patients will deliver more oral surgery services in a year [41].

State-level covariates were merged into the analytic file from the US Bureau of Labor Statistics, BRFFS, and IPUMS CPS, including the unemployment rate, the percentage of individuals with obesity, and the percentage of individuals reporting fair or poor health in a year, respectively, to adjust for general area-level differences in population wellbeing and health status. All models also included year and state fixed effects to adjust for secular time trends and time-invariant aspects of the Medicaid policies and other unique attributes of each state.

## Results

Table 1 summarizes the analytic sample. About 76.5% of our observations operated in Medicaid expansion states. Bivariate analyses demonstrated statistically-significant differences in both health center and state-level characteristics between the policy and comparison CHC groups. Across the full analytic sample, nearly half (48.4%) of the patients seen at the analytic sample CHCs lived below the poverty level in a year. About 9.7% and 8.8% of the patients were diagnosed with depression/mood disorder and diabetes mellitus in a year, on average.

**Table 1:** Characteristics of CHC-years analyzed from the pooled analytic sample, 2012–2017

	Full sample		Expansion-state CHCs		Non-expansion-state CHCs		
*Outcome characteristics*							
Oral surgery visits, per year	1051	(1417)	1067	(1330)	1001	(1677)	
*Health center characteristics*							
Hispanic patients, %	24.5%	(24.1)	26.0%	(24.6)	20.0%	(21.8)	**
White, non-Hispanic patients, %	43.7%	(29.2)	44.2%	(30.5)	41.9%	(24.4)	
Black, non-Hispanic patients, %	21.1%	(24.1)	18.5%	(23.0)	29.4%	(25.7)	**
Female patients, %,	56.4%	(6.7)	56.2%	(6.5)	57.1%	(7.1)	**
Patients < 18 years old, %,	26.5%	(12.8)	27.3%	(12.8)	23.6%	(12.5)	**
Patients < 100% of poverty level, %	48.4%	(24.4)	47.2%	(25.1)	52.7%	(21.3)	**
Patients with depression/mood disorder diagnosis, %	9.7%	(6.5)	9.9%	(6.6)	8.7%	(6.1)	**
Patients with diabetes mellitus diagnosis, %	8.8%	(4.2)	8.0%	(3.0)	11.4%	(6.1)	**
Total patients, in 1000 s	21.2	(23.8)	23.0	(25.8)	15.4	(14.1)	**
*State characteristics*							
Unemployment rate, %	4.2%	(0.8)	4.3%	(0.8)	4.0%	(0.7)	**
Population experiencing poor or fair health status, %	11.2%	(1.9)	11.0%	(1.8)	12.1%	(2.0)	**
Population experiencing obesity, %	28.4%	(3.5)	27.7%	(3.6)	30.8%	(1.5)	**
Observations (CHC-years)	2054		1572		480		

For each variable, unadjusted average percentages or totals per year are shown for CHCs in the analytic sample from 2012 to 2017. Standard deviations are shown in parentheses. Seventeen states were included in the analysis: AR, CO, IA, IL, MA, NC, NE, NM, NY, OH, OR, RI, SC, SD, VT, WI, WY. A ** denotes a statistically-significant difference in an observed variable between the expansion-state CHCs and non-expansion-state CHCs at the 0.01 level. *P* values were derived using two-sample t-tests, accounting for non-independent observations over time. Authors’ analysis of data from the Uniform Data System, Kaiser Family Foundation, Bureau of Labor Statistics, and IPUMS CPS

Table 2 shows the results of our multivariate analyses. The results of the event study model show that the likelihood of providing oral surgery services decreased relative to non-expansion-state CHCs following the Medicaid expansion. Compared to the year prior to expansion, expansion-state CHCs were 7.2% less likely than non-expansion-state CHCs to provide additional oral surgery services in 2015 (IRR = 0.928; *P* = 0.08) and 13.5% less likely in 2016 (IRR = 0.865; *P* = 0.06). By 2017, three years after the Medicaid expansion adoption, the expansion-state CHCs were 14.7% less likely to be providing oral surgery services on the margin relative to non-expansion-state CHCs (IRR = 0.853; *P* = 0.02). These results also provide statistical evidence suggesting the corollary of the common trends assumption discussed earlier was satisfactory: Before to the Medicaid expansion, the delivery of oral surgery services trended similarly across the policy and comparison CHC groups, as the pre-expansion event study coefficients did not statistically significantly differ from zero.

**Table 2:** Multivariate results estimating likelihood of delivering oral surgery following Medicaid expansion (n = 2054), 2012–2017

	Outcome: oral surgery services visits per year
*Difference-in-differences model*	
No expansion	Ref
Medicaid expansion × post-expansion	0.913*
	(0.034)
*Event study model*	
Medicaid expansion by time relative to expansion year	
2012 (Year −2)	0.998
	(0.052)
2013 (Year −1)	Ref
2014 (Year 0)	1.017
	(0.072)
2015 (Year 1)	0.928^+^
	(0.041)
2016 (Year 2)	0.865^+^
	(0.067)
2017 (Year 3)	0.853*
	(0.06)
*Covariates*	
Hispanic patients (%)	0.998
	(0.004)
White, non-Hispanic patients (%)	1.004
	(0.005)
Black, non-Hispanic patients (%)	1.002
	(0.004)
Female patients (%)	0.989
	(0.014)
Patients < 18 years old (%)	1.007
	(0.006)
Patients < 100% of poverty level (%)	0.998
	(0.004)
Patients with depression/mood disorder diagnosis (%)	0.991
	(0.009)
Patients with diabetes mellitus diagnosis (%)	0.966^+^
	(0.022)
Total patients (in 1000s)	1.018**
	(0.003)
Unemployment rate (state—%)	1.01
	(0.043)
Persons reporting poor or fair health (state—%)	1.004
	(0.009)
Persons experiencing obesity (state—%)	1.025*
	(0.012)

Authors’ analysis of data from the Uniform Data System, Kaiser Family Foundation, Bureau of Labor Statistics, and IPUMS CPS. ^+^*P* < 0.10, *P < 0.05, **P < 0.01. This table shows the event study coefficient estimates and the coefficient estimate from the difference-in-differences model. All multivariate parameter estimates are provided as incidence rate ratios (IRRs), or the cumulative incidence of an outcome in one group over the cumulative incidence of the outcome in the reference group (i.e., non-expansion-state CHCs), not as differences in the logs of expected counts. State effects and state year trend estimates not shown. Seventeen states were included in the main analysis, including AR, CO, IA, IL, MA, NC, NE, NM, NY, OH, OR, RI, SC, SD, VT, WI, WY

The results of the difference-in-differences model show that, relative to non-expansion-state CHCs, expansion-state CHCs included in the analytic sample were 8.7% less likely to provide oral surgery services on the margin in all post-expansion years pooled together (IRR = 0.913; *P* = 0.01), given the other variables in the model are held constant. Our coefficient estimate for the percentage of CHC patients diagnosed with diabetes mellitus in a year also appeared consistent with prior studies demonstrating inverse relationships between dental care use and diabetes diagnosis [39].

## Discussion

Our findings suggest that CHCs located in ACA Medicaid expansion states were less likely to deliver oral surgery services from 2014 to 2017 relative to non-expansion-state CHCs. These findings have implications for public policy and community oral health. Oral health is an important component of overall health status. Yet, in numerous developed counties, low socioeconomic status is linked to a greater burden of dental disease and unmet oral health care needs [3, 4, 42–44]. In the US, many low-income persons live in dental professional shortage areas [45] and cannot access oral surgery and other dental services when needed. CHCs aim to eliminate barriers to dental care. Historically, limited funding has constrained the amount of uncompensated dental care CHCs can provide [12], and it does not appear the ACA Medicaid expansion improved oral surgery treatment capacity at CHCs from 2014 to 2017.

These findings were unexpected. In addition to this study, Zwetchkenbaum and Oh [46] found that the percentage of Medicaid enrollees who received dental care at Rhode Island CHCs also decreased following the ACA Medicaid expansion. However, other recent studies have shown that the Medicaid expansion created opportunities for CHCs to expand Medicaid coverage in CHC patient populations [17], improve access to preventive medical services [16, 47], and expand patient revenue [18]. One explanation for our findings could be that expansion-state CHCs were infused with younger Medicaid-covered patients requiring less complex surgical services than non-expansion-state CHC patients. In their study of Medicaid expansion and dental care use among low-income adults, Singhal et al. [48] concluded that newly-Medicaid-covered childless adults may have been competing with traditional Medicaid enrollees for limited dental appointments following Medicaid expansion.

Alternatively, perhaps the expansion-state CHCs included in our study sample began to prioritize triaging complicated procedures to private dental providers with better treatment capacity and instead focused on providing lower-acuity preventive services to newly-covered patients from 2014 to 2017. CHCs often have limited dental treatment capacity, and other authors have cautioned that CHCs would be limited in their ability to make changes necessary to responding to increased demand for dental care following the Medicaid expansion [10]. Long-burdened by financial uncertainty [49], many CHCs likely find it challenging to identify and onboard new staff, prepare new facilities, and adapt their operations to accommodate greater patient demand [20]. On the other hand, CHCs—especially those that operate as Patient-Centered Medical Homes—actively coordinate care with other providers in the community, especially for patients who cannot easily navigate the medical and dental care systems [20]. Even when CHCs cannot afford to operate full dental practices, they often have the capacity to refer complex treatment needs to community partners accepting Medicaid-covered patients.

Cooperative efforts aside, other providers may have openly competed for newly-covered patients needing oral surgery following the Medicaid expansion. Only about 10% of CHCs operate in a full dental health professional shortage area, as designated by HRSA [50]. In some states, there is little financial incentive for private providers to provide dental services [46]. However, surgical services are typically reimbursed at higher rates by state Medicaid programs.

### Limitations

This study had several limitations. First, unobserved characteristics changing over time that are correlated with Medicaid expansion decisions and our outcomes may have biased our estimates (e.g., Medicaid benefit design reforms). We could not adjust for variation in patient need for dental services at CHCs. In theory, patient population demand for services could affect what types of services are offered by CHCs and whether state-level policymakers decide to expand Medicaid benefits for those populations. We did adjust for time-invariant unobserved policy and population characteristics at the state level to mitigate potential sources of omitted variable bias. We also adjusted for CHC-level differences in the prevalence of health conditions associated with oral health status and utilization as proxies for patient need.

Second, conclusions about changes to patient oral health outcomes or clinician care quality cannot be made using our estimates of grantee-level service delivery changes. Similarly, our grantee-level data could have masked the counteractive effects of patient movement in-and-out of CHCs, and we could only observe aggregate changes in average outcomes between the policy and comparison groups.

Third, this study sought to build upon the results of earlier studies examining CHCs in the states that first adopted the ACA Medicaid expansion in 2014, as well as to examine CHCs in states that cover adult oral surgery services through their Medicaid programs. For these reasons, though, the generalizability of our findings is limited to the states included the analytic sample. As a corollary of these limitations, it could be that the comparison CHCs identified in our analytic sample were located in states experiencing increases in the delivery of oral surgery services from 2014 to 2017 for either observable or unobservable factors eluding the study design.

## Conclusion

Although the ACA Medicaid expansion provided CHCs opportunities to gain more Medicaid-covered patients and expand patient revenue, our results from a large sample CHCS suggest that expansion-state CHCs operating in states that cover adult dental services through Medicaid were less likely to provide oral surgery services relative to non-expansion-state CHCs following Medicaid expansion in the years immediately following the policy. Relative to non-expansion-state CHCs, the expansion-state CHCs included in our study sample were 8.7% less likely to provide oral surgery services in all post-expansion years pooled together. Additional studies will be needed to better understand why these unexpected findings emerged, as well as to examine CHC dental treatment capacity following the ACA Medicaid expansion.

## Data Availability

The datasets used and analyzed during the current study are available from the U.S. Health Resources and Services Administration’s Health Center Program data repository, available through https://data.hrsa.gov/tools/data-reporting/program-data. These public use datasets are cited in the manuscript above.

## Abbreviations

ACA: Affordable Care Act
BRFSS: Behavioral risk factor surveillance system
BLS: Bureau of labor statistics
CDT: Code on dental procedures and nomenclature
CHC: Community health center
CPS: Current population survey
HRSA: Health Resources and Services Administration
IRR: Incidence rate ratio
UDS: Uniform data system
